# Impact of COVID-19 on pediatric emergency fellowship training in Saudi Arabia

**DOI:** 10.1186/s12245-023-00518-9

**Published:** 2023-08-28

**Authors:** Ahmad Khobrani, Osama Kentab, Abdulaziz Algarni, Ahmad AAl Ibrahim, Javid Ahmad Bhat, Ammar Abdulmajeed, Wafa Homaida, Sara El Basheer, Abdullah Akkam, Muna Aljahany

**Affiliations:** 1 Department of Emergency, King Abdullah bin Abdulaziz University Hospital, Princess Nourah Bint Abdulrahman University, Riyadh, Saudi Arabia; 2 King Saud Medical City, Riyadh, Saudi Arabia; 3 Department of Clinical Sciences, College of Medicine, Princess Nourah Bint Abdulrahman University, Riyadh, Saudi Arabia

**Keywords:** COVID-19, Medical education, Pediatric, Emergency fellowship

## Abstract

**Objectives:**

To assess the impact of the COVID-19 pandemic on the academic and clinical processes of pediatric emergency medicine (PEM) fellowship training held by the Saudi Commission for Health Specialties (SCHS).

**Methods:**

A cross-sectional, nationwide, survey-based study was conducted between June and December 2020. PEM program directors as well as fellowship trainees were eligible. The collected data were under the following domains: (1) sociodemographic and work-related characteristics; (2) impact of the COVID-19 pandemic on patient flow and PEM procedures; (3) impact on emergency skills and competence; (4) impact on academic performance; and (5) attitudes toward PEM practice and potential solutions. Monthly reports of PEM visits and procedures were also collected from program directors.

**Results:**

A total of 11 PEM program directors and 42 fellows responded. During the pandemic, the number of total ED visits decreased by 70.1%, ED inpatient admissions fell by 57.3%, and the number of intraosseous need insertion and lumbar puncture procedures fell by 76.7% and 62.3%, respectively; the temporal differences in the median frequencies were statistically significant. The pandemic has influenced the knowledge acquisition and leadership skills of one-third of program directors (36.4% and 27.3%, respectively) and the skills and competence of fellows (31.0%). The majority of directors and fellows showed that online classes/webinars were useful (100% and 95.2%, respectively), and there was no need to extend the current fellowship training to compensate for learning deficits (62.7% and 78.6%, respectively). The importance of dedicated modalities to fill in the training gap increased by 62.5% of program directors and 35.7% of fellows.

**Conclusion:**

The COVID-19 pandemic had significant effects on clinical procedures and academic activities in the PEM fellowship program. The impact was consistently perceived across PEM program directors and fellows. Technology-driven solutions are warranted to mitigate the expected learning and clinical deficits due to reduced clinical exposure.

## Introduction

The COVID-19 pandemic has dramatically influenced the behavior and actions of individuals, organizations, and communities worldwide, and health care organizations are no exception. Multiple operational changes have been made in hospitals, academic health centers, and medical practice institutions in response to the pandemic. For example, in Saudi Arabia, national authorities have undertaken measures to optimize health capacity and care for the surge in COVID-19 patients, extend services to private organizations, and instruct health care providers about the optimal use of personal protective equipment (PPE) [1–3]. The Ministry of Health has stressed the importance of implementing adequate hospital response measures, such as monitoring daily occupancy rates, reallocating isolation beds, transferring patients who do not experience COVID-19 symptoms to other hospitals, and suspending nonurgent and routine services in health care settings [4]. Furthermore, outpatient visits have transitioned to telemedicine [5], and clinical exposure has substantially decreased in multiple medical specialties. These pervasive changes have also entailed the redeployment of health care trainees to new roles, altered clinical exposure, and changes in targeted educational activities, such as lectures and conferences.

As a consequence, directors and leaders in academic medical education programs have adopted operational changes to respond to new clinical requirements and educational needs. Although scarce, data from Saudi Arabia revealed the effects of COVID-19 on residency and fellowship training programs for ophthalmology, internal medicine, and radiology [6–8]. However, little is known about the impact of these changes on pediatric emergency medicine (PEM) practice and training. The PEM fellowship program was established by the Saudi Commission for Health Specialties (SCHS) to improve the clinical experience of trainees in the management of critically ill children. This 2-year program helps achieve key competencies in triage, diagnosis, decision-making, case management, and follow-up care by providing a comprehensive academic curriculum and hands-on training sessions using models and/or equipment. PEM residents and trainees are required to be well prepared to deal with urgent cases in acute care facilities. Such capabilities are predominantly based on experience and clinical exposure. and hands-on training sessions might have been affected by shifts in health care modalities during the COVID-19 pandemic. The present study aimed to explore the impact of the COVID-19 pandemic on PEM fellow training programs across Saudi Arabia from the perspectives of program directors and trainees. The results of this study will help tailor a dedicated program with mitigation measures to compensate for the deficient aspects in the current program.

## Methods

### Study setting and ethical considerations

The study team at King Abdullah bin Abdul-Aziz University Hospital (KAAUH), which is located at Princess Nourah Bint Abdulrahman University (PNU), Riyadh, carried out a cross-sectional, survey-based study involving PEM program directors and fellows across the Kingdom of Saudi Arabia. The study was conducted between June and December 2020. The study protocol was approved by the institutional review board (IRB) of PNU [H-01-R-059#]. The collected data were kept confidential and were solely used for research purposes.

### Study population

The directors of the PEM program of the SCHS were eligible. In addition, first- and second-year PEM trainees who were enrolled during the post-pandemic period (from March to June 2020) were included. We excluded service PEM specialists who were not enrolled in the SCHS fellowship program and fellowship program trainees who were not enrolled in the post-pandemic period.

### Study procedure

Data collection was based on two main themes: (1) monthly reports of PEM visits and procedures as reported by program directors, and (2) survey-based data to collect self-perceptions of program directors and PEM fellows regarding the impact of COVID-19 on PEM fellowship training. Monthly PEM activities and procedures were collected for the period from March to June 2019 (the pre-pandemic reference period) and the same period in 2020 (during the pandemic) using dedicated data collection sheets on SurveyMonkey® (Momentive Inc.). These included the monthly number of PEM visits, the number of PEM inpatients admitted, and the number of the following PEM procedures: intubation, intraosseous needle insertion, and lumbar puncture. In addition, data on a number of resuscitation procedures were collected, including neonatal resuscitation, pediatric resuscitation, and major trauma resuscitation.

Two structured questionnaires were created on the SurveyMonkey® platform (one for program directors and another for PEM fellows). Survey items were retrieved from validated investigations [9–13], and the items were modified and adapted to be applicable in the context of the PEM fellowship program. Survey links were distributed to eligible participants via email and social media platforms.

The program director questionnaire comprised five domains (for the total 25 items listed in Appendix 1): (1) sociodemographic and work-related characteristics; (2) effects of the COVID-19 pandemic on patient flow and PEM procedures and duties; (3) effects of the COVID-19 pandemic on knowledge acquisition during shifts and emergency skills and competence; (4) effects of the COVID-19 pandemic on academic performance; and (5) attitudes toward PEM practice during the pandemic and potential solutions. The PEM fellow questionnaire consisted of five domains (for a total of 29 items listed in Appendix 2): (1) demographic, psychological, and COVID-19-related characteristics; (2) effects of the COVID-19 pandemic on PEM procedures and duties; (3) effects of the COVID-19 pandemic on emergency skills and competence; (4) effects of the COVID-19 pandemic on academic performance; and (5) attitudes toward PEM practice during the pandemic and potential solutions.

### Statistical analysis

Categorical variables were presented as frequencies and percentages, whereas continuous variables were demonstrated as medians and interquartile ranges (IQRs). The percent changes in ED visits between both pre- and post-pandemic periods were computed, and comparisons of numbers of ED visits were performed using the Mann–Whitney test. Statistical analysis was performed using the Statistical Package for Social Sciences version 26.0 (SPSS Inc., Chicago, IL, USA).

## Results

### Temporal changes in the number of visits, admissions, and selected procedural and managemental parameters across PEM training centers

**Fig. 1:**
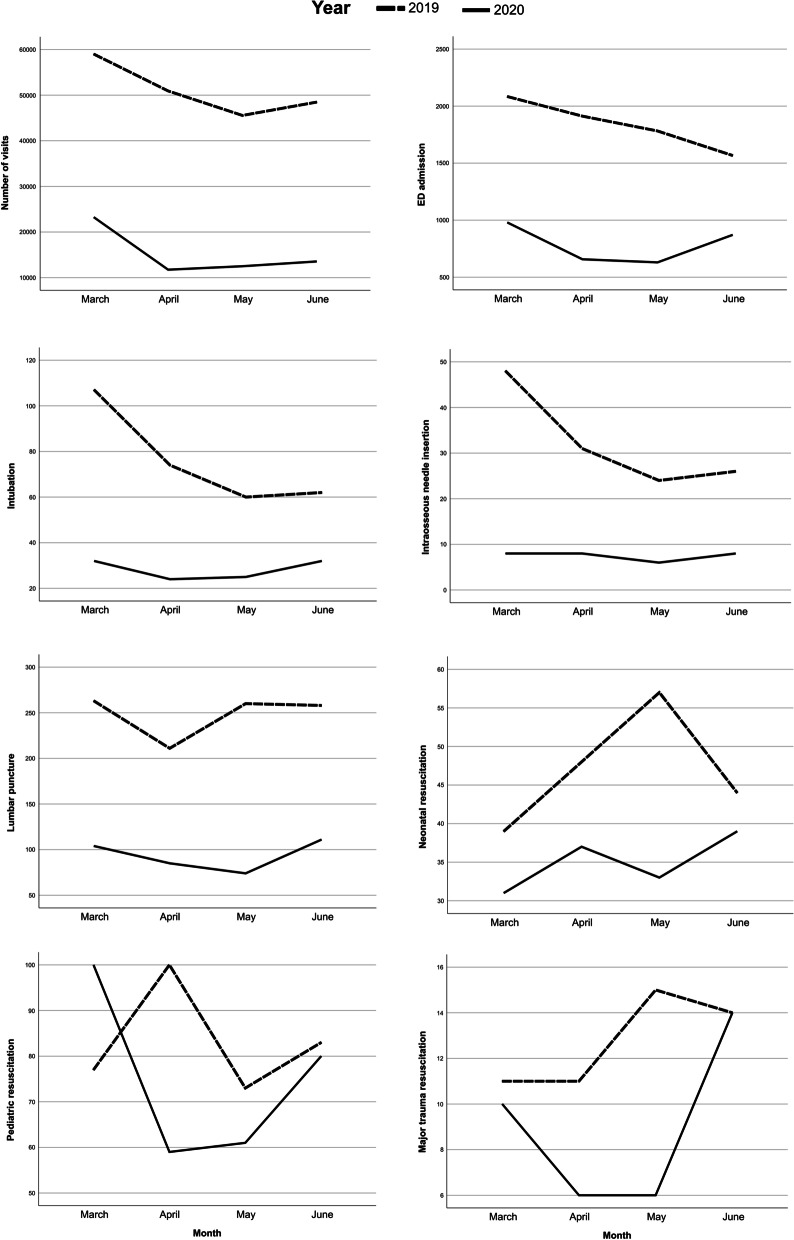
Temporal changes in selected parameters across PEM training centers

The changes in the total number of ED visits and admissions as well as the number of ED procedures and resuscitation interventions are shown in Table 1, and the monthly changes are visually depicted in Fig. 1. The total number of ED visits in 2020 was 61,048, which was 70.1% lower than that in 2019 (203,948 visits). Similarly, ED admissions fell by 57.3% from *n* = 7345 in 2019 to *n* = 3138 in 2020. The total number of emergent procedures performed at pediatric emergency departments has consistently dropped, with the largest decline for intraosseous needle access (76.7%), followed by intubation (62.7%) and lumbar puncture (62.3%). The differences between the pre- and post-pandemic periods were statistically significant for the median values of ED visits (*p* < 0.0001), ED admissions (*p* = 0.005), and two emergency procedures, including intraosseous needle access (*p* = 0.029) and lumbar puncture (*p* = 0.048). The number of resuscitation interventions showed modest reductions from March to June 2020, with no statistically significant temporal differences compared to the same month in 2019 (Table 1).

**Table 1:** Descriptive statistics of ED visits and admission as well as the pediatric emergency procedures and resuscitation interventions performed in the PEM training centers in selected months in 2019 and 2020

Parameter	Total number			Median (IQR)		
	2019	2020	∆n	2019	2020	p^*^
Total ED visits	203948	61048	70.1% ↓	5028.5 (1625–8616.5)	1361.5 (587.5–2247.5)	** < 0.001**
ED admission	7345	3138	57.3% ↓	224.5 (46.5–349.8)	90 (21.3–147.3)	**0.005**
Intubation	303	113	62.7% ↓	4.0 (2.0–17.0)	3.0 (1.8–6.0)	0.071
Intraosseous needle insertion	129	30	76.7% ↓	2.0 (0.8–7.3)	1.0 (0–2.0)	**0.029**
Lumbar puncture	992	374	62.3% ↓	14.0 (5.0–35.3)	6.5 (4.0–21.3)	**0.048**
Neonatal resuscitation	188	140	25.5% ↓	6.0 (1.0–12.0)	2.5 (0.39.0)	0.325
Pediatric resuscitation	333	300	09.9% ↓	6.0 (1.0–23.3)	3.5 (1.0–8.0)	0.296
Major trauma resuscitation	51	36	29.4% ↓	2.0 (0–4.0)	0.5 (0–3.5)	0.186

^*^Significant differences were assessed using the Mann–Whitney *U* test

### Responses of PEM program directors

#### Sociodemographic and work-related characteristics

A total of 11 PEM program directors responded to the survey. The respondents indicated that their training programs were primarily located in Riyadh (36.4%) or Jeddah (27.3%) and that the number of fellows enrolled in the programs ranged between 4 and 6 (54.5%) or less than 4 fellows (27.3%). Three program directors (27.3%) stated that their PEM departments had been converted to COVID-19 inpatient units to increase bed capacity. Furthermore, PEM fellows were redeployed from rotations to other PEM training centers or other planned pre-COVID-19 rotations, as indicated by 45.5% and 63.6% program directors, respectively.

#### The impact of the pandemic on patient flow and PEM procedures and duties

**Fig. 2:**
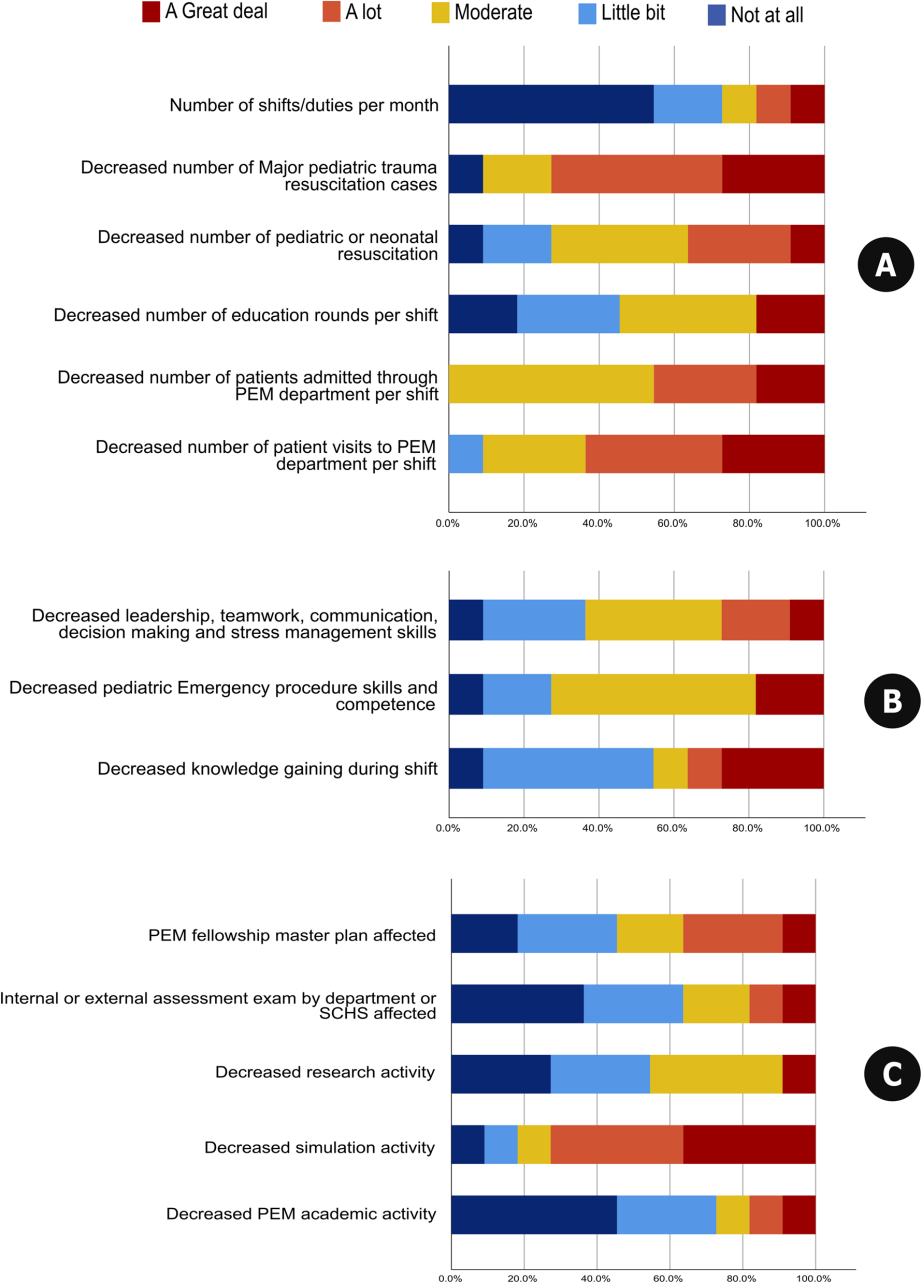
The responses of PEM program directors regarding the impact of the COVID-19 pandemic on selected parameters related to patient flow and PEM procedures and duties (**A**), knowledge acquisition during shifts and emergency skills and competence (**B**), and academic performance (**C**)

Regarding the impact of the COVID-19 pandemic on patient flow at PEM departments, the majority of program directors (who responded to the survey as “a lot” or “a great deal”) underscored a significant reduction in the number of major pediatric trauma resuscitation cases (72.8%) and the average number of visits to the PEM department per shift (63.7%, Fig. 2A).

### The impact of the pandemic on knowledge acquisition during shifts and emergency skills and competence

Regarding the aspects relevant to workload and competence, 36.4% of program directors stated that while the pandemic might have influenced knowledge acquisition during shifts, it has decreased leadership and teamwork skills as well as PEM procedural skills and competence, as revealed by 27.3% and 18.2% of respondents, respectively (Fig. 2B).

### The impact of the pandemic on academic performance

The most significant effects on academic performance were primarily related to reducing simulation activities (72.8%) and negatively affecting PEM fellowship master plans (36.4%), whereas research activities were minimally affected (9.1%, Fig. 2C).

#### Attitudes of program directors toward PEM practice during the pandemic and the potential solutions

Generally, the impact of the pandemic on PEM fellow training was rated as minimal to moderate by the program directors (as declared by 36.4% and 63.6% of respondents, respectively). Interestingly, online classes and webinars held during the lockdown period were appreciated by all respondents. Only one respondent agreed that trainees would not be able to achieve the optimal technical competence as an independent practitioner, while three participants (27.3%) felt that it is important to extend the current training period of mentorship for PEM fellows. In addition, eight program directors (62.5%) acknowledged the role of these proposed modalities to fill the training gap, particularly for extending simulation sessions (100%) and providing multiple hands-on courses (62.5%).

### Responses of PEM fellows

#### Demographic, psychological, and COVID-related characteristics

A total of 51 PEM fellows responded to the questionnaire; however, the responses of nine participants were excluded due to lack of primary outcomes. Out of the remaining respondents (*n* = 42), there were 24 males (57.1%), 26 first-year fellows (61.9%), and 30 participants aged 31–35 years (71.4%, Table 2). Most PEM fellows dealt with patients with a suspected or confirmed COVID-19 infection (97.6%), and thought that they had received adequate training on the proper use of personal protective equipment (PPE, 78.6%). Approximately three-quarters of PEM fellows (76.2%) declared that the pandemic had increased stress/anxiety levels, while 33.3% of fellows were satisfied with the psychological support provided by the SCFHS. Thirty-one participants (73.8%) were aware of the “DAEM” initiative intended for the psychological support of trainees during the pandemic; however, only two participants (4.8%) had enrolled in the program (Table 2).

**Table 2:** Demographic, psychological, and COVID-19-related characteristics of PEM fellows

Parameter	Category	Frequency	Percentage
Gender	Male	24	57.1
	Female	17	40.5
Age	25–30	11	26.2
	31–35	30	71.4
	36–40	1	2.4
Academic year	First-year fellow	26	61.9
	Second-year fellow	16	38.1
Province	Riyadh	27	64.3
	Jeddah	5	11.9
	Mecca	4	9.5
	Medina	1	2.4
	Asir	5	11.9
Do you manage patients with suspected or confirmed COVID-19 cases?	No	1	2.4
	Yes	41	97.6
Have you been infected by COVID-19?	No	39	92.9
	Yes	3	7.1
Have you been isolated from work as a suspected or confirmed case of COVID-19?	No	34	81.0
	Yes	8	19.0
Do you think the availability and training on PPE by your training center is adequate?	No	9	21.4
	Yes	33	78.6
How has the pandemic impacted your stress/anxiety levels?	Decreased	0	0
	No effect	10	23.8
	Increased	32	76.2
Are you satisfied with the psychological support provided to you by your training hospital and SCHS during the pandemic?	No	13	31.0
	Not sure	15	35.7
	Yes	14	33.3
Are you aware about the psychological support program “DAEM” offered by the SCHS?	No	9	21.4
	Yes, but not enrolled	31	73.8
	Yes, and I’m enrolled	2	4.8

*SCHS* Saudi commission for health specialties

#### The impact of the pandemic on patient flow and PEM procedures and duties

Regarding the impact of the pandemic on patient flow, eight participants (19.0%) indicated that the number of shifts/duties decreased during the COVID-19 pandemic. Approximately one-third of PEM fellows have an estimated a greater than 50% reduction in PEM visits (31.0%), and a more than 50% reduction in PEM procedures (35.7%) at the training centers.

#### The impact of the pandemic on emergency skills and competence

Approximately one-third of the PEM fellowship program trainees (31.0%) agreed that the pandemic has affected their PEM skills, competence, and performance. In addition, 19.0% of the trainees indicated that their teamwork, decision-making, and communication skills were affected.

#### The impact of the pandemic on academic performance

**Fig. 3:**
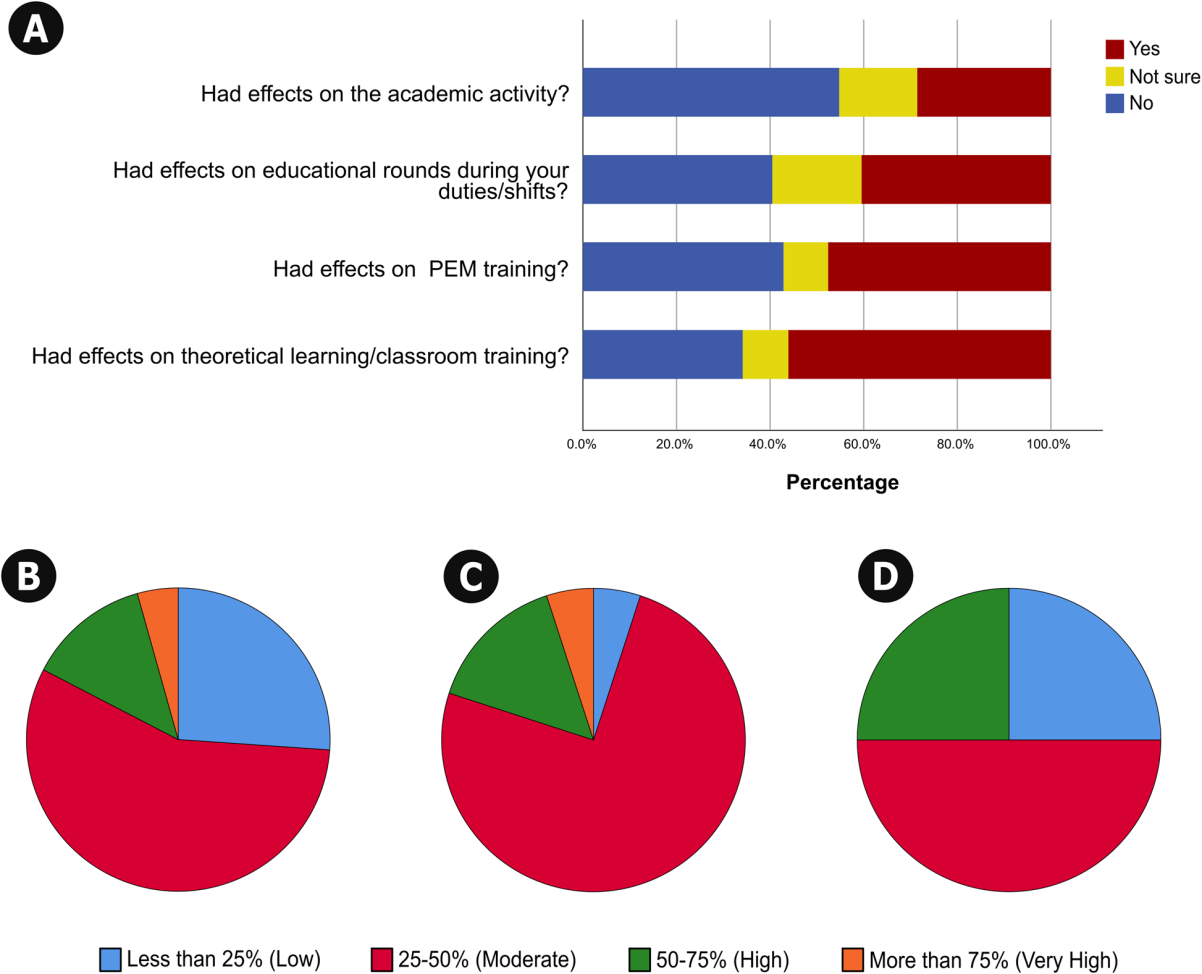
The responses of PEM fellows regarding their perceived effects of the COVID-19 pandemic on their academic activities and PEM training (**A**) and the degrees of negative effects on theoretical learning/classroom training (**B**), PEM fellowship training (**C**), and PEM fellowship academic activities (**D**)

Concerning academic performance, 12 PEM fellows (28.6%) perceived negative effects on their academic activities in the post-pandemic period. Theoretical learning, PEM training, and educational rounds during shifts were the most negatively affected, as indicated by 56.1%, 47.6%, and 40.5% of the participants, respectively (Fig. 3A). Among those who perceived negative effects on academic performance, the effects were rated “high” to “very high” by 17.3% of participants for theoretical learning/classroom training (Fig. 3B), 20.0% for PEM fellowship training (Fig. 3C), and 25.0% for PEM fellowship academic activities in general (Fig. 3D). Interestingly, 31 PEM fellows (73.8%) indicated a negative influence of COVID-19 on didactical and practical training activities, which impacted self-professional growth.

#### Attitudes of PEM fellows toward PEM practice during the pandemic and the potential solutions

**Fig. 4:**
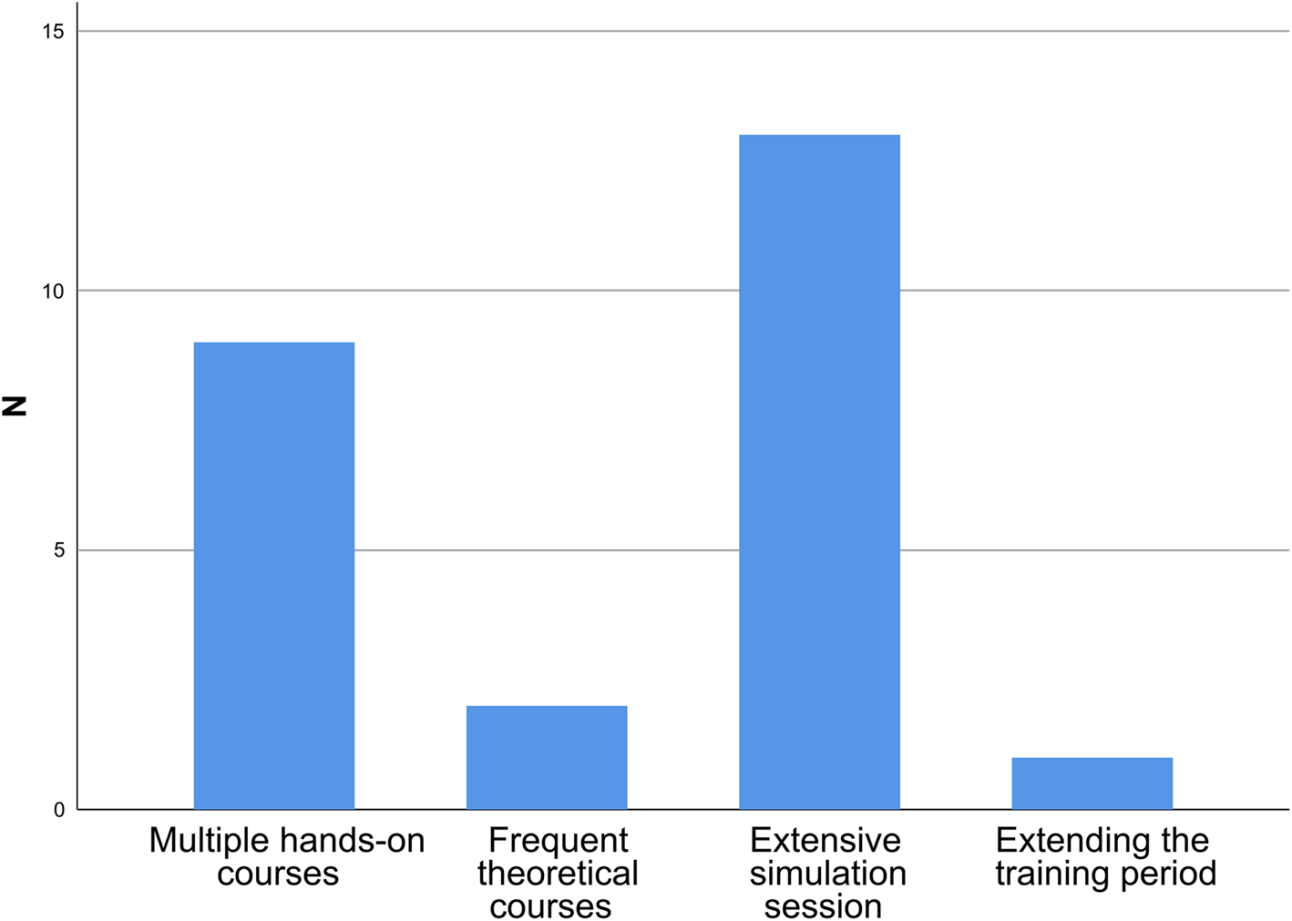
The responses of PEM fellows regarding the proposed solutions to address the training gap

A large proportion of respondents agreed that online classes and webinars were useful during the lockdown period (95.2%) but indicated that there was no urgent need to extend the training period or the period of mentorship to compensate for the negative effects of COVID-19 on PEM training (78.6%). To fill the training gap, 15 PEM fellows (35.7%) corroborated the importance of conducting distinct modalities during the training. Of them, 13 (86.7%) and 9 (60.0%) participants acknowledged the impact of extensive simulation sessions and multiple hands-on courses to address the training gap (shown in Fig. 4).

## Discussion

The global COVID-19 pandemic has caused significant changes in various clinical settings, resulting in iterative programmatic changes in the academic medical niche. Consistent with the increased burden among adults compared with children, pediatric emergency departments responded differently than adult emergency units. In our study, the number of PEM visits plunged by 70.1%, and ED admissions plunged by 57.3%. In New York City, Sagalowsky et al. [14] also reported a significant reduction in PEM volume by 50–80% due to the shutdown of elective and nonessential procedures and the conversion of clinical spaces into intensive care units (ICUs) and adult COVID-19 departments. The authors indicated that PEM fellows in three different programs were redeployed to ICUs, medicine inpatient units, and adult emergency departments [14]. The present analysis also showed that the frequencies of lumbar puncture and intraosseous access procedures were greatly reduced in the post-pandemic period; this trend may be attributed to concerns about the highly contagious nature of the virus. The loss of volume in cases may be academically detrimental for fellows, particularly for those with case-volume requirements. Therefore, future plans should be tailored to meet the graduating criteria, whether by having a case-by-case competence assessment program for individual fellows or by extending the fellowship period [15].

Concerning the responses of PEM directors and fellows, we showed similar patterns of self-perception regarding the post-pandemic reduction of PEM flows and procedures as well as the respondents’ attitudes toward the necessity of online classes/webinars. Additionally, approximately three-quarters of directors and fellows agreed that there is no need to extend the training period of PEM mentorship. While rapidly evolving circumstances have influenced the leadership and teamwork skills of program directors, the pandemic has also impacted the skills and performance of fellows. Compared to fellows, a higher proportion of program directors (62.5% versus 35.7% for program directors and fellows, respectively) appreciated the role of alternative modalities to fill in the gap in fellows’ training.

The findings of this study are generally in line with other findings in the literature regarding the negative effects on the learning process in fellowship programs. Indeed, approximately half of PEM fellows reported a significant impact on theoretical learning, PEM training, and educational rounds during shifts. In a recent study involving medical residents and fellows in Saudi Arabia, the majority of fellows (85.2%) and senior residents (81.8%) indicated a reduction in training activities during the pandemic [8]. Additionally, similar to our findings, more than half of the fellows felt stressed, anxious, or worried about the situation during the pandemic [8]. This is in agreement with the fact that depression and anxiety are commonly reported among medical staff of first-line units, such as emergency departments, infectious diseases, and intensive care units, who are frequently in close contact with potentially infected patients [16]. Furthermore, the psychological burden is prominent among junior staff, fellows, and residents who are not prepared with appropriate mental health care training and not equipped with relevant coping strategies [17, 18].

Of note, approximately one-third of PEM directors (36.4%) stated that knowledge acquisition has significantly reduced during the post-pandemic period. Furthermore, a considerable proportion of fellows indicated a 50% reduction in PEM training activities, which affect their skill development, competence, and performance. Similarly, the majority of ophthalmology trainees in multiple countries reported a 50% reduction in clinical activity and a more than 75% reduction in surgical activities [19].

In this study, the importance of online classes and webinars was emphasized by all program directors and most fellows. Indeed, the pandemic has caused many training programs to modernize their educational content and update their method of delivery in a way that is familiar to residents and fellows. For example, in the USA, videoconferencing has been used as an alternative to on-site visits that had originally been utilized in selecting an otolaryngology fellow or fellowship programs [20]. A working group of the Coalition for Physician Accountability also recommended that all fellowship programs should commit to virtual visits and online interviews for all students for the entire residency interview cycle [21].

Importantly, extensive simulation sessions and hands-on courses were suggested by approximately two-thirds of PEM directors and one-third of fellows as potential solutions to COVID-19-related learning deficits in the present analysis. Likewise, homemade and online simulation models, surgical videos, and webinars have been proposed as sources for important skill training for obstetrics and gynecology trainees, as indicated by a recent systematic review of the relevant literature [22]. The positive attitudes of program directors and fellows toward online solutions and simulation paradigms reflect the importance of these technology-driven methods in different fellowship processes to guarantee the continuity of education and ensure a sustained learning curve for fellows [23].

The present study has some limitations. Variation in the perceptions of PEM program directors and fellows regarding different outcomes was not statistically assessed because the questions were not consistent in either the director or fellow questionnaires. For example, program directors were asked to rate their perceptions regarding the volume of PEM visits, whereas fellows responded to a question regarding a more than 50% reduction in PEM visits. The limited number of respondents might have also impacted the robustness and reliability of the data. Consequently, the implications of our results may not be generalizable to other regional settings. Finally, the cross-sectional nature of our study might have been affected by response bias in which some respondents may have accidentally or intentionally provided false responses.

In conclusion, the COVID-19 pandemic had devastating effects on patient flow and the frequency of inpatient admission to PEM departments, with significant declines in distinct emergency procedures, including intraosseous needle access and lumbar puncture. Self-reported stress/anxiety levels were high among the majority of PEM fellows, indicating a need for psychological support via dedicated multidisciplinary health care teams that involve social workers, psychiatrists, and other mental health workers. PEM program directors and fellows reported similar patterns of self-perception regarding post-pandemic changes in patient volume at PEMs and knowledge and skills acquisition during shifts. Both groups equally appreciated the importance of online classes/webinars, and the majority of them showed no need to extend the training period to compensate for learning deficits. A higher proportion of program directors acknowledged the importance of implementing corrective modalities, especially extensive simulation sessions and hands-on courses, to fill training gaps. Future large-scale, longitudinal studies are required to corroborate the negative impact of reduced clinical exposure and academic activities during fellowship training. Smart technology should be widely adopted in national fellowship programs to account for the expected slowdown of PEM fellows’ learning curve in the post-pandemic period.

## Data Availability

The datasets generated during and/or analyzed during the current study are available from the corresponding author on reasonable request.

## Appendix 1

The domains, items, and the available choices of the PEM director questionnaire.

**Table:**

Domain	Item	Response(s)				
Sociodemographic and work-related characteristics	Your training center?	Riyadh	Jeddah	Mecca	Medina	Asir
	How many fellows are currently enrolled in your Fellowship Program?	< 4	4–6	6–8	> 8	
	Has any part of the PEM department been converted to inpatient COVID 19 units as a part of the efforts to increase Capacity?	No	Yes			
	Has any of your PEM fellows been redeployed from rotation to other PEM training centers as a joint program?	No	Yes			
	Has any of your PEM fellows been redeployed from rotation to other planned pre-COVID-19 rotation?	No	Yes			
Effects of the COVID-19 pandemic on patient flow and PEM procedures and duties	Decreased number of patient visits to PEM department per shift	Not at all	Little bit	Moderate	A lot	A Great deal
	Decreased number of patients admitted through PEM department per shift	Not at all	Little bit	Moderate	A lot	A Great deal
	Decreased number of education ٌrounds per shift	Not at all	Little bit	Moderate	A lot	A Great deal
	Decreased number of pediatric or neonatal resuscitation	Not at all	Little bit	Moderate	A lot	A Great deal
	Decreased number of major pediatric trauma resuscitation cases	Not at all	Little bit	Moderate	A lot	A Great deal
	Number of shifts/duties per month	Not at all	Little bit	Moderate	A lot	A Great deal
Effects of the COVID-19 pandemic on knowledge acquisition during shifts and emergency skills and competence	During the pandemic COVID-19 period, how do you rate the following: decreased knowledge gaining during shift	Not at all	Little bit	Moderate	A lot	A Great deal
Effects of the COVID-19 pandemic on the academic performance	During the pandemic COVID-19 period, how do you rate the following: emergency procedure skills and competence	Not at all	Little bit	Moderate	A lot	A Great deal
	During the pandemic COVID-19 period, how do you rate the following: decreased leadership, teamwork, communication, decision making and stress management skills	Not at all	Little bit	Moderate	A lot	A Great deal
Attitudes towards PEM practice during the pandemic and the potential solutions	During the pandemic COVID-19 period, how do you rate the following: Decreased PEM academic activity	Not at all	Little bit	Moderate	A lot	A Great deal
	During the pandemic COVID-19 period, how do you rate the following: decreased simulation activity	Not at all	Little bit	Moderate	A lot	A Great deal
	During the pandemic COVID-19 period, how do you rate the following: decreased research activity	Not at all	Little bit	Moderate	A lot	A Great deal
	During the pandemic COVID-19 period, has internal or external assessment exam by department or SCHS affected	Not at all	Little bit	Moderate	A lot	A Great deal
	During the pandemic COVID-19 period, has PEM fellowship master plan affected?	Not at all	Little bit	Moderate	A lot	A Great deal
d.attitude,	Were online classes and webinars during the lockdown period useful?	No	Yes			
	With cancelling of non-urgent cases across many institutions during the COVID 19 pandemic, how much impact do you feel the current crisis will have on the training of your current class of PEM fellows?	No	Minimal to mild	Moderate	Severe	
	Are you concerned that at least 1 of your current fellow trainees will be not able to achieve the technical competence to successfully perform their roles as an independently practicing PEM trainee next year?	No	Yes			
	Do you feel that there should be an extension of the training period or mentorship for the current class PEM fellows?	No	Yes			
	Do you think proposed modality to fill in the training gap such as multiple hands on courses, increase the number of theoretical courses and extending the training period will be a valuable thing for trainees?	No	Yes			
	If yes, as PEM program director, which mitigation program will be valuable for PEM trainees?	Frequent theoretical courses	Extensive simulation session	Extending the training period	Multiple hands-on courses	

## Appendix 2

The domains, items, and the available choices of the PEM fellow questionnaire.

**Table:**

Domain	Item	Response(s)				
Demographic, psychological, and COVID-19-related characteristics	Gender	Male	Female			
	Age	25–30	31–35	36–40	41–45	
	Currently working as	First year fellow (F1)	Second year fellow (F2)			
	Your training centers?	Riyadh	Jeddah	Mecca	Medina	Asir
	Do you manage patients with suspected or confirmed COVID-19 cases?	No	Yes			
	Have you been infected by COVID-19?	No	Yes			
	Have you been isolated from work as a suspected or confirmed case of COVID-19?	No	Yes			
	Do you think the availability and training on PPE by your training center adequate?	No	Yes			
	How do you think during the pandemic COVID-19 period, lockdown, social distancing has affected your stress/anxiety level?	Decreased	No effect	Increased		
	Are you satisfied with the psychological support provided to you by your training hospital and SCfHS, during pandemic COVID19 and lock down period?	No	Not sure	Yes		
	Are you aware about psychological support program DAEM offer by Saudi commission for health specialties for training HealthCare worker?	No, I did not know about them	I know about them, but I didn’t get in touch with them	Yes, I am enrolled in the program		
Effects of the COVID-19 pandemic on PEM procedures and duties	During the pandemic COVID-19 period, lockdown, and social distancing, how much did pediatric emergency visits decrease in your training center?	Dropped by < 25%	Dropped by 25–50%	Dropped by 50–75%	Dropped by > 75%	
	During the pandemic COVID-19 period, lockdown, social distancing and decrease PEM department visits, how much did pediatric emergency procedures decrease in your training department?	Dropped by < 25%	Dropped by 25–50%	Dropped by 50–75%	Dropped by > 75%	
	The Number of your shifts/duties during pandemic COVID-19 crises and lockdown has	Decreased	Same	Increased		
Effects of the COVID-19 pandemic on emergency skills and competence	Had pandemic COVID-19 affected your emergency procedure skills, competence, and performance as a fellowship trainee?	No	Not sure	Yes		
	Had pandemic COVID-19 affected your leadership, teamwork, communication, decision making and stress management as PEM fellowship trainee?	No	Not sure	Yes		
Effects of the COVID-19 pandemic on the academic performance	Do you think during the pandemic COVID-19 has affected your PEM training?	No	Not sure	Yes		
	If yes, how much of a negative effect on your PEM fellowship training?	Less than25%	25–50%	50–75%	More than75%	
	Do you think during the pandemic COVID-19 period, lockdown, social distancing and decreasing PEM department visits has affected your theoretical learning/classroom training?	No	Not sure	Yes		
	If yes, how much of a negative effect do you think pandemic COVID-19 period, lockdown, social distancing and decrease PEM department visits had on theoretical learning/classroom training?	Less than 25%	25–50%	50–75%	More than75%	
	The pandemic COVID-19 have affected your educational rounds during your duties/shifts?	No	Not sure	Yes		
	The pandemic COVID-19 affected your academic activity?	No	Not sure	Yes		
	If yes, how much of a negative effect do you think pandemic COVID-19 period, lockdown, social distancing and decrease PEM department visits had on your PEM fellowship academic activity?	Less than 25%	25–50%	50–75%	More than 75%	
	How has the didactic academic activity offered by your training center changed during COVID-19?	We have no change in our academic activity schedule	Academic activity is reduced compared to pre-COVID	Academic activity is the same compared to pre-COVID	Academic activity is increased compared to pre-COVID	
Attitudes towards PEM practice during the pandemic and the potential solutions	The decrease in the training activity (both didactical and practical) will affect your training and professional growth?	No	Yes	A little	A lot	
	Were online classes and webinars during this lockdown period useful?	No	Not sure	Yes		
	Do you think there should be an extension of your training period or mentorship due to the impact of COVID19 on your training?	No	Not sure	Yes		
	Do you think as a PEM fellow trainee proposed modality to fill in the training gap such as multiple hands on courses, increasing the number of theoretical courses and extending the training period will be a valuable thing for you?	No	Not sure	Yes		
	if yes, as a PEM fellow, which mitigation program will be valuable for you?	Frequent theoretical courses	Extensive simulation session	Extending the training period	Multiple hands-on courses	
